# Effect of the PREPARE intervention on sexual initiation and condom use among adolescents aged 12–14: a cluster randomised controlled trial in Dar es Salaam, Tanzania

**DOI:** 10.1186/s12889-017-4245-4

**Published:** 2017-04-17

**Authors:** Elia John Mmbaga, Lusajo Kajula, Leif Edvard Aarø, Mrema Kilonzo, Annegreet Gera Wubs, Sander Matthijs Eggers, Hein de Vries, Sylvia Kaaya

**Affiliations:** 1 Department of Epidemiology and Biostatistics, Muhimbili University of Health and Allied Sciences, PO Box 65015, Dar es Salaam, Tanzania; 2 Department of Psychiatry and Mental Health, Muhimbili University of Health and Allied Sciences, Dar es Salaam, Tanzania; 3 Department of Health Promotion and Development, Faculty of Psychology, University of Bergen, Bergen, Norway; 4 Department of Health Promotion, Norwegian Institute of Public Health, Bergen, Norway; 5 Department of Health Education and Health Promotion, Maastricht University, Maastricht, The Netherlands

## Abstract

**Background:**

Unsafe sexual practices continue to put adolescents at risk for a number of negative health outcomes in Tanzania. While there are some effective theory-based intervention packages with positive impact on important mediators of sexual behaviours, a context specific and tested intervention is urgently needed in Tanzania.

**Purpose:**

To develop and evaluate an intervention that will have a significant effect in reducing sexual initiation and promoting condom use among adolescents aged 12–14 in Dar es Salaam, Tanzania.

**Design:**

A school-based Cluster Randomised Controlled Trial was conducted during 2011–2014 in Kinondoni Municipality.

**Methods:**

A total of 38 public primary schools were randomly selected, of which half were assigned to the intervention and half to the control group based on their size and geographic location. Participants were interviewed using a self-administered questionnaire at baseline before the PREPARE intervention and then, 6 and 12 months following intervention. The primary outcomes were self-reported sex initiation and condom use during the past 6 months. Data analysis was done using Generalized Estimating Equation (GEE) modelling controlling for repeated measures and clustering of students within schools.

**Results:**

A total of 5091 students were recruited at baseline, and interviewed again at 6 (*n* = 4783) and 12 months (*n* = 4370). Mean age of participants at baseline was 12.4 years. Baseline sociodemographic, psychometric and behavioural characteristics did not significantly differ between the two study arms. The GEE analysis indicated that the intervention had a significant effect on sexual initiation in both sexes after controlling for clustering and correlated repeated measures. A significantly higher level of action planning to use condoms was reported among female adolescent in the intervention arm than those in the control arm (*p* = 0.042). An effect on condom use behaviour was observed among male adolescent (*p* = 0.004), but not among female (*p* = 0.463).

**Conclusions:**

The PREPARE intervention had an effect in delaying self-reported sexual initiation among adolescents aged 12–14 in Dar es Salaam Tanzania. The intervention positively influenced action planning to use condoms for both sexes and increased actual condom use among male adolescents only. Future interventions addressing adolescent sexual and reproductive health should focus on impacting mediators of behaviour change.

**Trial registration:**

Australian New Zealand Clinical Trials Registry ACTRN12613000900718, registered on 13 August, 2013.

## Background

The prevalence of HIV infection among young people in Tanzania remains high with female adolescents (4.4%) having twice as high HIV prevalence as compared to their male counterparts (1.7%) [1]. While efforts to provide HIV education for prevention are scaled up, only 40% of young people aged 15–24 have comprehensive HIV knowledge. Data indicates practice of risky sexual behaviours, such as unprotected sex (42%) and multiple sexual partnerships (14%), among sexually active young people are widespread [1].

Studies that have examined effects of interventions based on the Social Cognitive Theory indicate that sexual and reproductive behavioural interventions among young people are effective, especially when targeting young adolescents before they become sexually active [2]. Such interventions produce more impact when they are broad and target many aspects of adolescent’s life including school and home environments, policies related to health, and adolescent wellbeing [3]. Our previous South Africa and Tanzania (SATZ) intervention in Dar es Salaam was successful in delaying self-reported sexual initiation among school aged adolescents, strengthening the notion that school-based interventions may be effective in changing young people’s behaviours [4]. Similar to the intervention evaluated in this study (PREPARE - Promoting sexual and reproductive health among adolescents in southern and eastern Africa – mobilising schools, parents and communities), the SATZ intervention addressed a range of social cognition factors. The SATZ intervention however, may have relied rather heavily on a teacher-centred delivery approach with less peer and health sector involvement in skill building. This limitation may have contributed to lack of effects in skill driven behaviours such as condom use. In this study, we developed and evaluated an intervention named PREPARE addressing adolescents’ risky sexual and reproductive behaviours for the purpose of delaying sexual debut and increasing consistent condom use among sexually active adolescents, which may reduce unwanted pregnancies and transmission of HIV and other sexually transmitted diseases. This intervention intended to have multiple components which aimed at impacting key mediators of the behaviour change process such as self-efficacy, social norms, attitude and action planning. This paper presents results of the effects of the PREPARE intervention in delaying early sexual initiation and promoting condom use among school-based adolescents aged 12–14 years in Dar es Salaam, Tanzania.

## Methods

### Design

This was a prospective cluster randomized controlled trial that tested the effects of the PREPARE intervention on reducing sexual initiation and increasing condom use among adolescents aged 12–14 years in Dar es Salaam. Details of the design and conduct of this study are presented elsewhere [5]. The study involved a baseline survey followed by the actual intervention implemented over a period of 9 weeks. A follow up survey was then conducted during month 6 then a booster (shorter version of the intervention) followed by a 12-month final follow up survey. In the PREPARE study, schools were set as the unit of randomization. Random assignment of schools to study groups rather than individual classrooms decreases the possibility of cross-group contamination among students in the same school, which would have threatened the study’s internal validity. School selection and randomization was done before baseline data collection.

### Sample size estimation

The sample size calculation was based on our previous estimate of sexual initiation of 4.8% [4] and aimed at reducing this estimate to 3.0% (37.5% reduction) following the intervention. To be able to achieve 80% power with a significance level of 0.05, and taking into account inter-cluster correlation, 19 intervention schools and 19 control schools were needed (Total 38 schools) giving an estimated total of 5090 adolescents were needed [5].

### School selection and randomization

A total of 38 schools from a list of 115 eligible public primary schools in the Kinondoni Municipality of Dar es Salaam were selected for this study. Twenty-three schools were not eligible and removed from the list due to being recently established and lacking students in standard 5 and 6, being privately owned, hence catering for a higher socio-economic status group than public schools, if they were included in the pilot phase of the project or were schools for disabled students. A pairwise block randomization with random selection of 38 schools based on the school size (number of students) and location (urban or peri-urban) was done. Location selection was proportional to the number of schools in urban or peri-urban areas. For each of the 19 pairs of schools, one school was randomly allocated to the intervention group, and the second, the control group using computer generated randomization.

### Prepare intervention

Details of the intervention are presented in our previous publication [5]. In short, the intervention consisted of three components, one implemented by teachers, one by peer educators, and one was implemented by health care providers during adolescents’ visits to youth friendly health service clinics. Three peer-led lessons taught over 8 h and six teacher-led lessons taught over 11 h were conducted. The teacher led lessons were integrated in the primary school science curriculum and taught as 16 interactive teaching and learning sessions suited for large classes with some didactic lessons, each session lasting for 40–80 min. Peer-led lessons were implemented over 9 weeks (once a week), each session lasting 60–90 min. The sessions which were part of an after-school life skills training curriculum, were designed to be interactive and teachers were available to offer support when needed.

The third component intended to link adolescents to information and services that may foster healthy sexuality. This component also promoted collaboration between schools and youth friendly health services, and increased the possibilities for future access to sexuality and reproductive health information and services by young adolescents with needs. Visits to health facilities exposed adolescents to existing services; a condom use demonstration session with male and female models allowed adolescents to handle male and female condoms and demonstrate their correct use, and feedback sessions following the visits were used to gain views from adolescents on their experience of the services offered by the clinics.

Assessment of intervention fidelity was done through observations and facilitators diaries. Members of the study team observed and rated approximately 25% of the teachers’ intervention sessions using a fidelity observation instrument that was developed by the study evaluation team.

### Participants and data collection procedures

All adolescents aged 12–14 in the selected schools were eligible and invited to participate in the study. Inclusion criteria included adolescents of both genders, able to read and write, of any ethnicity/race or socio economic status, who assented participation; with a custodial parent/guardian who gave consent by not objecting to their adolescent’s participation after receiving an information letter for the study. Of the parents/guardians who received the information letter, less than 3% refused and the main reason being fear that participating in the project may result into the child understanding about sex which may lead into an intention to experiment. Participants were selected from all standard 5 and 6 streams in the intervention and control schools. Most schools had a maximum of two streams of standard 5 and 6. All students who were eligible and had parental/guardian consent participated after given their written assent.

Data were collected using a self-administered paper questionnaire that was developed based on formative assessment and literature review of existing scales in the field. These included the HAVES scale for economic status and scales for the measurement of action planning to use condoms or to delay sex, social norms to use condoms and to delay sex, attitudes towards delay of sexual debut and towards condom use, self-efficacy to delay sex and to use condoms, communication with peers, friends and parents, myths about condoms, and puberty knowledge. The developed questionnaire was pilot tested in a sample of 400 adolescents and repeated after 4 weeks. The questionnaire collected information on socio-demographic characteristics of the participants such as age, sex, class level, living arrangements at home, religion, parental education, HIV prevention knowledge, attitudes, and action planning; as well as sexual health communication with peers and parents as behavioral and social cognition determinants of the behaviors of interest. Outcomes of interest were sexual initiation (reported any vaginal or anal intercourse during the past 6 months) and extent of practice of protected sex (reported condom use during the last vaginal or anal intercourse). Measures that were scaled (for example attitudes towards delaying sex and condom use) were subsequently evaluated on convergent validity (associations with other scales or variables of interest), internal consistency (Cronbach’s alpha). Test-retest correlations were assessed by calculating the intra-class correlation coefficient (ICC) for interval data, or by Cohen’s kappa for categorical data). The questionnaire was developed in English, translated into Swahili and independently back-translated into English. The original English version was compared with the back-translated version, and the Swahili version was revised before being administered. The questionnaire was administered in class under supervision of project staff without the presence of teachers. Repeated callbacks were done to schools with a large number of missing participants. Data were collected at baseline before the intervention, and 6 and 12 months following the intervention.

All participants were given unique identification numbers at baseline and this number was written in the baseline questionnaire. The participant was given an envelope with his/her unique identification number which was produced during follow up before completing the 6 and 12 months follow up questionnaires. No names were written on the questionnaires. The investigators kept a master list of all the numbers to ensure that participants produce their original assigned numbers during follow up. All participants who participated in the follow up surveys kept their original numbers.

### Data analysis

Data were checked for completeness and consistency and later double entered to ensure quality and cleaned using the Statistical Package for the Social Sciences (SPSS for Windows version 18). All inconsistencies were verified and cleared with the responsible data collector. STATA version 13 was used for statistical analysis. Categorical variables were summarized using proportions and differences between proportions and significance testing was carried out with adjusted Wald F-test. Means and standard deviations for continuous baseline variables were calculated and differences between means examined using a Wald’s F-test with adjustment for cluster effects. Confirmatory factor analysis was used to identify latent factors (using the Kaiser criterium: eigenvalue >1 and Oblimin rotation). Items with factor loadings lower than 0.40 were omitted from inclusion in a scale. The incidence of sex initiation was calculated after 6 and 12 months of follow up. All reported sexual initiations during the follow up period were considered incident cases and time at sexual initiation assumed to be at the mid-follow up time. We examined the appropriate correlation structure for the repeated measure analysis; the best structure was chosen using Quasi-Likelihood Information Criterion (QIC) as a measure of goodness of fit in Generalized Estimating Equation modelling (GEE). GEE models were built to examine changes in the mean score of various scales over time for the intervention and control groups while controlling for the correlation emanating from repeated measures and clustering of students within schools. Robust standard errors were estimated. GEE is an extension of the generalized linear model widely used in the analysis of longitudinal correlated data where valid robust standard errors based on quasi-likelihood theory are applied as opposed to the maximum likelihood theory for independent observations. All significance testing was two-tailed and significance level was set at 5%.

### Ethical considerations

This study was reviewed and ethically approved by the Muhimbili University of Health and Allied Sciences Ethics Review Committee and by the Western Norway Regional Committee for Medical and Health Research Ethics. Permission was also granted by the Kinondoni Municipal Education Department and the authorities of participating schools. Written informed consent was sought from parents/guardians of all participants. All eligible adolescents were given an information sheet about the project and two copies of a consent form to take to their parents.

Adolescents were asked to return one signed copy of the consent form from their parents/guardian before participation. All adolescents whose parents/guardians consented for participation were then requested to grant their assent before participation. Those agreeing to give written assent were recruited for this study. All the data were handled with great confidentiality and no names appeared in the questionnaire used for data collection. The trial was registered by the Australian New Zealand Clinical Trials Registry ACTRN12613000900718 (Dar es Salaam).

## Results

A total of 5091 adolescents aged 12 to 14 from 38 public primary schools in Kinondoni Municipality in Dar es Salaam were recruited in this study. Of those participated, 2503 (49.2%) were from randomly selected intervention schools and the rest from the randomly selected control schools. The overall mean age was 12.41 (standard deviation 0.62) years, with adolescents in the control schools not significantly older than those in intervention schools [mean age 12.43 (std. 0.63) versus 12.39 (std. 0.61); *p* = 0.3467, respectively].

**Fig. 1:**
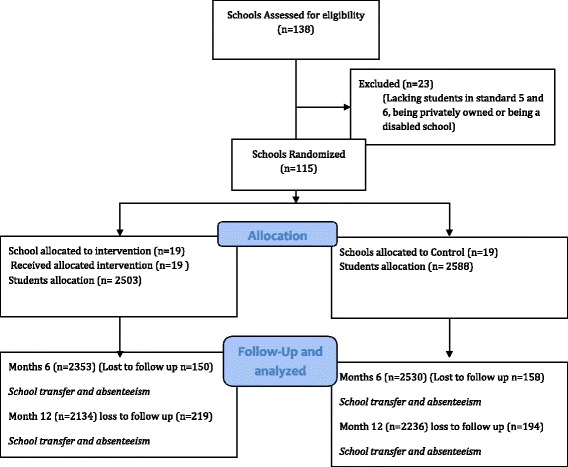
Diagrammatic presentation of participant recruitment, follow up and lost to follow up over time

Participants in both arms were followed for 12 months with data collection waves at baseline, 6 and 12 months. The proportion lost to follow up is displayed in Fig. 1. After 6 months, a total of 308 (6.0%) participants were lost to follow up and at 12 months, 721 (14.2%) of participants were lost to follow up. This loss to follow up at 12 months did not differ significantly by intervention status. The main reasons for the loss to follow up were students’ transfer to schools outside the study area and absenteeism.

### Comparison of baseline parameters

Comparison of socio-demographic characteristics by intervention status is presented in Table 1. Socio-demographic characteristics and school performance did not statistically differ between the intervention and the control arm (Table 1).

**Table 1:** Cluster adjusted comparison of baseline socio-demographic characteristics of study participants by intervention status

Variable	Category	Control	Intervention	*P*-value
Age	12	1679 (64.9)	1691 (67.6)	
	13	701 (27.1)	645 (25.8)	
	14	208 (8.0)	167 (6.7)	0.4538
Sex	Male	1267 (49.7)	1251 (49.1)	
	Female	1282 (50.3)	1260 (50.9)	0.7543
Class	5	583 (33.9)	902 (36.8)	
	6	1660 (66.1)	1547 (63.2)	0.2494
Religion	Christian	1315 (51.2)	1233 (49.5)	
	Muslim	1253 (48.8)	1259 (50.5)	0.7198
Mothers education	No/primary incomplete	241 (9.7)	234 (9.6)	
	Primary	743 (29.8)	678 (27.9)	
	Secondary	504 (20.2)	508 (20.9)	
	Above secondary	423 (17.0)	442 (18.2)	
	Don’t know	582 (23.3)	570 (23.4)	0.8703
Fathers education	No/primary incomplete	177 (7.3)	172 (7.3)	
	Primary	501 (20.7)	466 (19.7)	
	Secondary	522 (21.5)	484 (20.5)	
	Above secondary	569 (23.5)	569 (24.1)	
	Don’t know	657 (27.1)	672 (28.4)	0.8999
School performance	Good performance	2291 (91.1)	2225 (91.7)	
	Poor performance	223 (8.9)	202 (8.3)	0.3072

Baseline cluster adjusted mean scale comparisons of various psychometric and behavioural characteristics between intervention and control arms are presented in Table 2. Scores on psychometric and behavioural scales did not differ significantly between the two study arms.

**Table 2:** Cluster adjusted baseline comparison of mean scores between the intervention and control groups

Variable	Intervention	Control	Mean difference	*P*-value
Self-efficacy	2.266	2.225	0.0407	0.3074
Peer communication	1.646	1.606	0.0394	0.3216
Communication with friends	1.384	1.338	0.0465	0.0984
Communication with parents	1.338	1.305	0.0335	0.1222
Self-efficacy to delay sex	2.591	2.596	−0.0046	0.8879
Self-efficacy to use condoms	2.439	2.479	−0.0396	0.2356
Social norms condom use	3.352	3.407	−0.0549	0.2994
Social norm delayed sex	3.516	3.487	0.0297	0.5492
Attitude delayed sex (negative)	2.321	2.297	0.0239	0.5017
Attitude delayed sex (positive)	3.433	3.433	0.0002	0.9844
Action plan condom use	2.598	2.578	0.0198	0.4743
Action plan delayed sex	2.809	2.793	0.1529	0.6163
Puberty knowledge	1.504	1.503	0.0005	0.9862
Myths about condoms	2.231	2.244	−0.0127	0.7087
Haves	3.771	3.992	−0.2208	0.3687

### Sexual activity and incidence of sexual initiation

At baseline, 10.7% of participants from the intervention arm and 8.9% from the control arm reported to be sexually active and this achieved statistical significance at the 5% level (*p* = 0.026). During the follow up period, the rate of sexual initiation was estimated both for the intervention and the control arm. Table 3 depicts the rate of sexual initiation by sex and intervention status among adolescents aged 12–14 in Dar es Salaam. For males, the incidence of sexual initiation in the intervention arm decreased from 11.5/100 person years at risk (PYAR) at month 6 to 7.3/100 PYAR at the 12 month follow up. For the control arm, the incidence of sexual initiation at 6 months was 13.2/100 PYAR and that at 12 months was 10.9/100PYAR. The rate of sexual initiation at 12 months following the PREPARE intervention was almost 2 times higher among the control schools as compared to the intervention schools [Adjusted Relative Risk (ARR) = 1.9, *p* = 0.027].

**Table 3:** Change in the incidence of sexual initiation following the PREPARE intervention among adolescents aged 12–14 in Dar es Salaam, Tanzania

Follow up time	Intervention			Control		
Males						
	N	n	I	N	n	I
6 months	1158	65	11.5	1213	78	13.2
12 months	1158	87	7.3	1213	126	10.9
			*ARR = 1.9, p = 0.027*			
Females						
6 months	1052	60	11.7	1113	52	9.6
12 months	1052	75	7.4	1113	114	10.7
			*ARR = 1.6, p = 0.019*			

A similar analysis for female adolescents revealed the incidence of sexual initiation to have changed from 11.7/100 PYAR to 7.4/100 PYAR at month 6 and 12, respectively for the intervention arm. An increase in the incidence of sexual initiation was observed in the control arm changing from 9.6/100 PYAR to 10.7/100 PYAR from month 6–12, respectively. As it was the case for males, females from the control arm were 1.6 times more likely to initiate sex as compared to those from the intervention arm (ARR = 1.6, *p* = 0.019) (Table 3).

### Effect of the PREPARE intervention on action planning and actual behaviour

Extended generalized estimating equation modelling was performed to examine the effect of the PREPARE intervention on the mean score for action planning and actual behaviour variables while controlling for clustering and repeated nature of the observations collected from the same individuals over time.

Results show that there was an increase in the mean score for action planning to delay sex and condom use among males during the period of 12 months of follow up for both intervention and control arm with no significant difference between intervention and control arm (Table 4). Males in the intervention group were less likely to report sexual initiation at the end of the study as compared to those in the control group (*p* = 0.043). Moreover, a significantly large increase in reported condom use was reported by males in the intervention as compared to the control arm (*p* = 0.004).

**Table 4:** Cluster adjusted effects of the PREPARE Intervention on action planning and actual sexual behaviours among adolescents aged 12–14 in Dar es Salaam, Tanzania

		INTERVENTION		CONTROL	
Variable	Occasion	Coefficient	*p*-value	Coefficient	*p*-value
Males					
Action plan to delay sex	Baseline	ref	ref	ref	ref
	Month 6	0.0729	0.3713	0.1049	<0.001
	Month 12	0.1497	<0.001	0.1562	<0.001
	*Group*	*0.0254*		*P = 0.614*	
Action plan to use condom	Baseline	ref	ref	ref	ref
	Month 6	0.1629	0.0325	0.2956	0.014
	Month 12	0.1880	0.0191	0.2772	0.043
	*Group*	*0.0740*		*P = 0.0876*	
Sexual initiation	Month 6	0.1249	1	0.0272	1
	Month 12	0.0656	0.007	0.0192	0.684
	*Group*	*-0.1126*		*P = 0.043*	
Condom use	Baseline	ref	ref	ref	ref
	Month 6	0.3209	<0.001	0.1484	0.025
	Month 12	0.3694	<0.001	0.2672	<0.001
	*Group*	*0.2173*		*p = 0.004*	
Females					
Action plan to delay sex	Baseline	ref	ref	ref	ref
	Month 6	0.1469	0.025	−0.1830	0.046
	Month 12	0.1055	0.033	0.1539	0.067
	*Group*	*0.0967*		*P = 0.0873*	
Action plan to use condom	Baseline	ref	ref	ref	ref
	Month 6	0.1724	<0.001	0.1916	<0.001
	Month 12	0.1537	0.0412	0.0857	0.176
	*Group*	*0.1801*		*P = 0.042*	
Sexual initiation	Month 6	0.148	ref	0.0694	ref
	Month 12	0.0746	<0.001	0.1318	<0.001
	*Group*	*-0.1361*		*P = 0.009*	
Condom use	Baseline	ref	ref	ref	ref
	Month 6	0.1727	<0.001	0.2660	<0.001
	Month 12	0.2910	<0.001	0.3070	0.001
	*Group*	*0.0162*		*P = 0.463*	

**Fig. 2:**
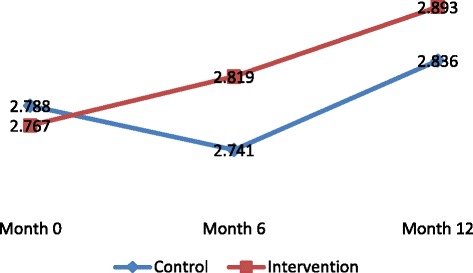
Change overtime in adjusted mean scale for action planning to delay sex among Female adolescents

**Fig. 3:**
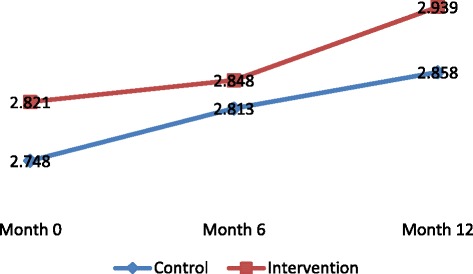
Change overtime in adjusted mean scale for action planning to delay sex among Male adolescents

**Fig. 4:**
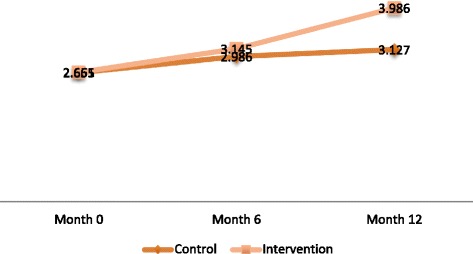
Change in adjusted mean scale overtime for action planning to use condom among Male adolescents

**Fig. 5:**
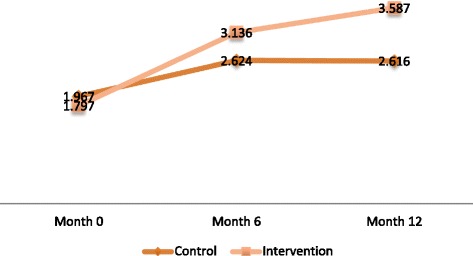
Change in adjusted mean scale overtime for action planning to use condom among Female adolescents

With regards to female adolescents, a significant increase in action planning for condom use (*p* = 0.042) and decreased reporting of sexual initiation (*p* = 0.009) were observed among those in the intervention arm as compared to the control arm (Table 4, Figs. 2, 3, 4 and 5).

## Discussion

The PREPARE intervention was designed to promote delayed sexual initiation and safer sexual behaviours among adolescents [5]. The intervention was developed based on findings from previous studies in the geographical area and the PREPARE formative phase, making it population specific and culturally sensitive. Implementation of the intervention was rigorous and well monitored with a high degree of fidelity to facilitate attribution of observed changes to the intervention.

The intervention was found to have a positive effect in reducing sexual initiation for both male and female adolescents in Dar es Salaam. The effect was observed immediately after the intervention and sustained over 12 months. These findings on sexual debut are similar to what was reported in the earlier intervention in Dar es Salaam during the SATZ study [4]. A systematic review of school based interventions to reduce HIV risk concluded that intensive behavioural interventions reduced sexual HIV risk, especially because they increased skill acquisition, sexual communications, condom use, delayed onset of sexual intercourse, and the number of sexual partners [6]. While recent reviews have indicated that education curricula alone may not be effective in inducing behavioural change, the intensive and multifaceted intervention programme used in this study that covered teacher centred class room teaching, peer-education and youth friendly clinic visits may partly explain the observed positive change [7]. It is worth noting that, although safer sex is a human right, reduction in sexual activity among young adolescents of this age (12–14 years) is taken to be a positive aim for preventive interventions. This is due to the fact that at this age sexual organs may be immature, sexual negotiation may be lacking, and sexual activity may have detrimental consequences.

Positive change in the level of action planning to delay sex for both males and female was observed over time in this population. The effect of the intervention on this underlying mediator of sexual initiation might have played an important role in the observed actual behaviour. On one hand, these findings are corroborated by results from various other studies that indicate cognitions such as beliefs, attitudes, subjective and descriptive norms, perceived behavioral control, self-efficacy, intentions and action planning do predict behavior, and those interventions that are at least partly based on social cognition models succeed in influencing behavior in sub-Saharan Africa [8, 9]. On the other hand, one review did not find effects of behavioral interventions on condom use. This lack of evidence of effect was assumed to be due to poor quality of studies including high loss to follow up [10].

Action planning to use condoms improved among adolescents in this study. The changes were stronger in the intervention arm as compared to the control arm. These changes may be attributed to the observed positive effects on condom use among male adolescents but not among female adolescents. The observed positive effect on the condom use behaviours among males could be due to the fact that male condoms are the most available; use of which is most centred on a male partner’s decision to use. Condom use among females in African cultural setting, on the other hand may also require successful negotiation with a male partner, despite of favourable action planning for use on her part. This observation supports an increasing understanding that the broad nature of interventions through addressing different contextual factors may produce better effects. Interventions should address personal factors such self-efficacy and action planning, and interpersonal factors such as communication with partners to be able to achieve the desired effect [3]. As opposed to the SATZ intervention, the PREPARE intervention involved access to youth friendly services as a skill building component for correct use of condoms. Visits to these clinics helped to bridge the gap between school-based theoretical training and actual skills development training conducted by health care workers as per the Tanzanian policy and guidelines [11].

The results of this study emanate from a rigorously designed randomised controlled trial with a high degree of intervention fidelity, an attribute for high quality research needed to inform policy. The strength of our intervention is its’ basis in theory, with a formative phase that allowed for gathering locally generated evidence, and collaborative inputs from stakeholders (teachers, health care providers, parents and learners) that were reflected in the content and approaches for implementation of this study. In the future development of intervention and scaling up, more emphasis should be given to tailoring, local adaptability and the multifaceted nature of the intervention to address broader areas of concern to bring about desirable behavioural change.

Substantial efforts dedicated by the research team to recruit, follow and maintain research participants contributed to lower loss to follow up, giving the study good external validity. However, the results of this study may have been limited by the fact that all behavioural estimates were based on participant’s self-reports, which could be liable to desirability bias. Random allocation of schools to the different study arms with consequent excellent comparability of the intervention and control arms as well as the small random attrition rates observed study provide strengths to the results presented.

## Conclusions

We conclude that the PREPARE intervention was successful in delaying sexual initiation among male and female adolescents aged 12–14 years in Dar es Salaam Tanzania. The intervention positively influenced action planning to use condoms for both males and females (significantly for females), but impacted actual reported condom use only among male adolescents. Future interventions addressing adolescent sexual and reproductive health issues should be culturally sensitive and adopt broader approaches which impact underlying mediators of behavioural changes to be able to facilitate actual behavioural change.
